# Design and Development of Nanomaterial-Based Drug Carriers to Overcome the Blood–Brain Barrier by Using Different Transport Mechanisms

**DOI:** 10.3390/ijms221810118

**Published:** 2021-09-19

**Authors:** Jisu Song, Chao Lu, Jerzy Leszek, Jin Zhang

**Affiliations:** 1 School of Biomedical Engineering, University of Western Ontario, 1151 Richmond Str., London, ON N6A 5B9, Canada; jsong254@uwo.ca; 2 Department of Chemical and Biochemical Engineering, University of Western Ontario, 1151 Richmond Str., London, ON N6A 5B9, Canada; clu248@uwo.ca; 3 Department of Psychiatry, Wroclaw Medical University, Pasteura 10, 50-367 Wroclaw, Poland; jerzy.leszek@umed.wroc.pl

**Keywords:** blood–brain barrier, nanomaterials, brain drug delivery, BBB viral disruption, transnasal route

## Abstract

Central nervous system (CNS) diseases are the leading causes of death and disabilities in the world. It is quite challenging to treat CNS diseases efficiently because of the blood–brain barrier (BBB). It is a physical barrier with tight junction proteins and high selectivity to limit the substance transportation between the blood and neural tissues. Thus, it is important to understand BBB transport mechanisms for developing novel drug carriers to overcome the BBB. This paper introduces the structure of the BBB and its physiological transport mechanisms. Meanwhile, different strategies for crossing the BBB by using nanomaterial-based drug carriers are reviewed, including carrier-mediated, adsorptive-mediated, and receptor-mediated transcytosis. Since the viral-induced CNS diseases are associated with BBB breakdown, various neurotropic viruses and their mechanisms on BBB disruption are reviewed and discussed, which are considered as an alternative solution to overcome the BBB. Therefore, most recent studies on virus-mimicking nanocarriers for drug delivery to cross the BBB are also reviewed and discussed. On the other hand, the routes of administration of drug-loaded nanocarriers to the CNS have been reviewed. In sum, this paper reviews and discusses various strategies and routes of nano-formulated drug delivery systems across the BBB to the brain, which will contribute to the advanced diagnosis and treatment of CNS diseases.

## 1. Introduction

Central nervous system (CNS) diseases, such as dementia, Alzheimer’s disease and gliomas, are the leading causes of disability and death [1,2,3,4]. However, the effective strategies on the treatment of these CNS diseases have not yet been developed because only very few drugs with a small molecular weight could cross the blood–brain barrier (BBB) and keep their activities in the CNS [2,5].

Thus, it is critically important and an urgent demand to understand the BBB transportation and have efficient strategies to deliver drugs through crossing the BBB. Recently, nanomaterial-based drug delivery systems have been studied by exploiting physiological BBB transport (transcytosis mechanism). Inorganic and organic nanostructures with surface modification have been designed as drug carriers to cross the BBB. In addition, during the period of the COVID-19 pandemic, the studies on neurotropic viruses, their BBB disruption mechanisms and their invasion methods to the CNS are vital to fight the global crisis. Meanwhile, their BBB disruption and/or circumvention mechanisms may give an inspiration on developing innovative drug delivery systems for the treatment of these CNS diseases.

In this paper, the structure of the BBB and its physiological transport mechanisms are introduced, followed by the introduction of the viral disruption mechanism. Nanomaterial-based drug carriers with enhanced capability for BBB transportation are reviewed based on different transport mechanisms (carrier-mediated, adsorptive-mediated and receptor-mediated transcytosis). Furthermore, nanocarriers by exploiting viral-induced BBB disruption mechanisms are discussed, which is considered an alternative method to cross the BBB and could be the next-generation drug vehicles. Finally, we discuss various delivery routes of nano-formulated drugs to the brain.

## 2. Crossing the BBB by Transportation and Disruption

### 2.1. Structure of the BBB

**Figure 1:**
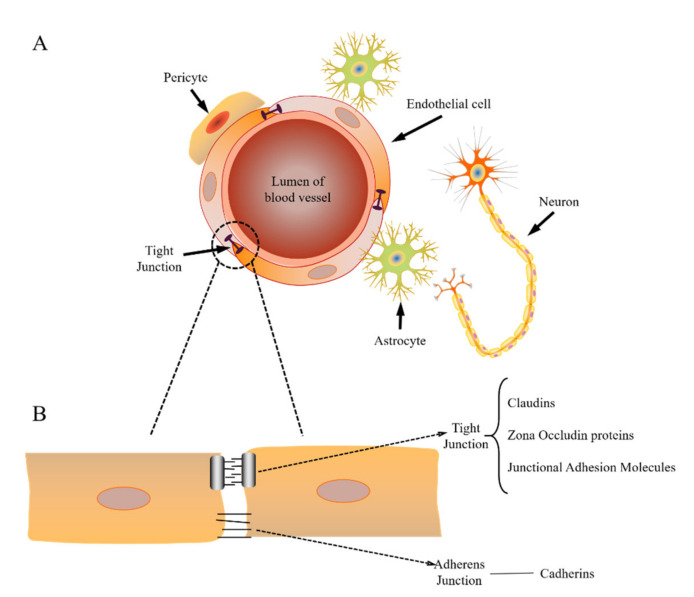
(**A**) Schematic of structure and components of the BBB. (**B**) Structure of endothelial intercellular junctions (including tight junctions (TJs) and adherens junctions (AJs)).

The BBB is one of the most extensive and exclusive physical barriers that maintain homeostasis within the CNS by protecting it from toxins and metabolic fluctuations [6]. The BBB is mainly composed of endothelial cells with other supporting structures, including brain capillaries, pericytes, astrocytes, and the basement membrane [5,7,8,9,10] (Figure 1A). These structures work together to supply the brain with the required nutrients, such as glucose and oxygen, for normal neural functioning, while preventing neurotoxins from entering the neural cavity. Because the brain is tightly packed with micro-vasculatures, the neuronal cells are in close proximity to blood capillaries. One of the differences between the capillaries of the BBB compared to capillaries found elsewhere in the body is the lack of fenestration in the endothelial cells [9]. The lack of fenestration prevents the passive diffusion of hydrophilic substances crossing the BBB through paracellular transport. Furthermore, the endothelial cells are tightly packed then connected through adherens junctions (AJs) and tight junctions (TJs) (Figure 1B) [5,8,9]. AJs provide the tissue structural support and hold the cells together. Cadherin proteins span the intercellular cleft and are linked into the cell cytoplasm [8]. Junctional adhesion molecules (JAMs) and the intercellular-cleft-spanning proteins (occludin and claudins) form the complex structure of TJs, the latter of which are linked to the regulatory proteins, including zona occludens (ZO) (ZO-1, ZO-2, ZO-3) and cingulin [8,11]. Moreover, the electrical resistance of brain endothelium is much higher than other endothelial cells, which introduces even further limitations in paracellular transport [9]. Due to these structural limitations, almost all substances could not cross the BBB via transcellular transport. There are few exceptions, namely, very small molecules (less than 400 Da) and lipophilic molecules, which can diffuse through the lipid bilayer of the cell membrane [9].

Major physiological BBB transport mechanisms are passive diffusion, carrier-mediated transcytosis (CMT), adsorptive-mediated transcytosis (AMT) and receptor-mediated transcytosis (RMT). However, nanomaterials, with the advantages of a high drug-loading capacity, good stability, biodegradability, low toxicity and versatility, make good drug transportation systems across the BBB and make the delivery of the loaded drugs into the CNS possible [6,12,13,14,15,16]. The physical and chemical properties, shape, size, hydrophobicity, and surface charge of nanomaterials can be controlled to ‘disguise’ themselves to mimic the molecules or particles that could cross the BBB, resulting in the enhancement of BBB transport and theragnostic agent’s treatment efficiency. Therefore, new strategies and drug delivery systems based on nanomaterials have been developed to treat brain diseases in recent decades.

At the same time, some therapy strategies by using mechanical, ultrasound and chemical agents were able to disrupt BBB and aid the transportation of drugs across the BBB. However, such disruption was reported to cause severe vasculopathy, chronic neuropathologic changes and seizures in animal models [5]. It is worth noting that other BBB breakdown strategies, in addition to the factitious way mentioned above, have existed in some viral infections (such as HIV-1) and their induced CNS diseases. Such disruption is probably caused by the direct effects of microbial products or the indirect effects on the tight-junction-related proteins [11]. Thus, understanding the mechanism of the viral disruption of the BBB would provide a new horizon on the BBB transportation and developing an innovative drug carrier system.

### 2.2. Physiological Transport Mechanisms

**Figure 2:**
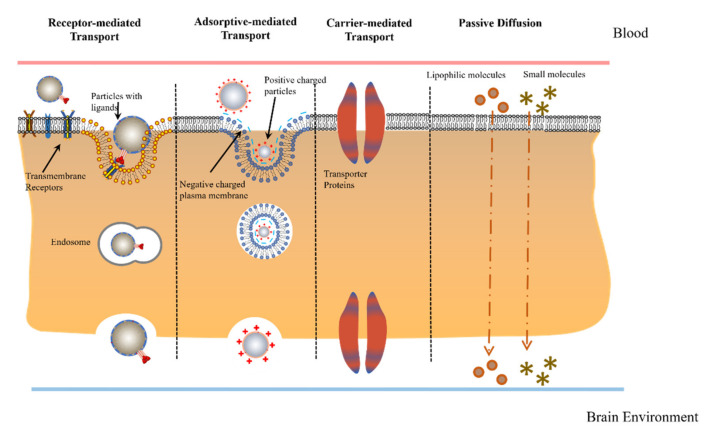
Physiological transport mechanisms crossing the BBB.

Substances cross the BBB by following one of the four transport mechanisms: passive diffusion, carrier-mediated transport, adsorptive-mediated transcytosis, and receptor-mediated transport (Figure 2). Passive diffusion is the transport mechanism that can only be used by the lipid-soluble small molecules mentioned earlier. These molecules freely diffuse across the BBB by lipid-mediated diffusion. There are not many known substances that use this transport mechanism, as the size limitation as well as the necessity to be lipophilic are uncommon characteristics [9].

Carrier-mediated transport is one of the most common transport mechanisms. Substances enter the endothelial cells via their corresponding transmembrane proteins on the cell membrane. An example would be glucose transportation via glucose transporter type 1 (GLUT1). GLUT1 is able to recognize glucose, mannose and galactose and actively transport these substances through the BBB [17]. Another example is phenylalanine transport via large neutral amino-acid transporter type 1 (LAT1). LAT1 is able to transport phenylalanine, as well as ten other large neutral amino acids, through the BBB [18]. It is also able to transport some neutral amino acids but to a lesser extent. Furthermore, LAT1 has been used in drug delivery systems. L-DOPA, a drug for Parkinson disease, is also able to successfully cross the BBB via LAT1 [18].

Adsorptive-mediated transcytosis (AMT) results from the electrostatic interaction between the positively charged ligands and the negatively charged cell membrane. It is mediated by clathrin-dependent endocytosis and is unidirectional from blood to brain [9]. Receptor-mediated transport (RMT) is the other most common transport mechanism. Instead of having transmembrane transporter, peptide receptors on the cell membrane mediate transcytosis of the ligands. This mechanism works for blood-to-brain transport, brain to blood transport, and blood-to-brain capillary endothelium transport without the export into the brain parenchyma [9].

### 2.3. Viral Disruption Mechanism

In addition to the physiological transport mechanisms, many viruses, such as Japanese encephalitis virus (JEV), human immunodeficiency virus-1 (HIV-1) and rabies virus, are found to be able to infect the CNS by other mechanisms and may cause severe neurologic syndromes. Flavivirus, coronavirus and other neurotropic viruses are briefly introduced, and their possible disruption mechanisms are also reviewed (Table 1) and discussed below. The disruption of the BBB is both a cause and effect of viral-induced CNS diseases [11]. The investigation on such viral-induced BBB breakdown mechanisms may contribute to developing new strategies to overcome the BBB. 

Flaviviruses are major emerging human pathogens, and some of the flaviviruses, such as West Nile virus (WNV), Japanese encephalitis virus (JEV), Dengu virus and Zika virus, are regarded to invade the CNS by various mechanisms [40,41]. During the flavivirus infection, the disruption of the BBB has been largely evidenced in the experimental models [42]. The invasion mechanisms are under investigation and are speculated to be various. Hepatitis C virus (HCV) entry receptors were observed on brain microvascular endothelia and brain endothelial cells, which support the entry and replication of the virus [19]. JEV and WNV invade the CNS by increasing the expression of matrix metalloproteinases 9 (MMP9) [20,21]. Such protein is related to the degradation of the basement membrane and the cleavage of tight junction proteins occludin and claudin-5, leading to BBB dysfunction [11].

In addition, HCoV-OC43 and HCoV-229E are two wild-spread coronaviruses and are proved to be neuro-invasive [43] and neurotropic [28]. The HCoV-229E could mainly be spread to the CNS under an immune-suppressed environment [30], and such neuro-invasion of this virus is mainly dependent on the circulation of bloodstream [28], by the facilitated passage of infected monocytes/macrophages towards the CNS [44]. On the other hand, the penetration of HCoV-OC43 could also be neuronal retrograded [28] apart from the hematogenous pathway [29]. Such dissemination could start from the olfactory bulb to the cortex and the hippocampus [45], and the virus-induced increased cytokine production may generate glutamate excitotoxicity and neuronal degeneration [28]. Besides, a preliminary study also showed that the RNA of HCoV-OC43 could be detected in the CNS of infected mice and would be persistent for one year [46].

SARS-CoV-2 is a SARS-like single-stranded RNA coronavirus with 29,903 bp [47], and has high genetic similarity with SARS-CoV (79.5%) and bat coronavirus RaTG13 (97%) [24,48]. Similar to other coronaviruses, such as SARS-CoV, spike glycoprotein (S-proteins) on the viral surface is able to bind to the cell membrane, followed by the infection of host cells. Recent studies showed that S-proteins of SARS-CoV and SARS-CoV-2 both have affinity to human angiotensin-converting enzyme 2 (hACE2) [24,25,26], which is expressed in the lungs, heart, kidneys, intestines and brain cells [47,49], although SARS-CoV-2 has around 10–20-fold higher affinity [27] and it shares different binding sites [24,50] on interaction with the ACE2 receptor. ACE2 is a typical zinc metallopeptidase, which could regulate blood pressure, and is proved to be expressed in the non-cardiovascular and cardiovascular areas of brain nuclei [51]. The high affinity of this virus to ACE2 may gain its ability to enter and infect cells in the CNS via hematogenous or neuronal retrograde dissemination [28]. This virus can not only mainly contribute to the symptoms, such as fever, dry cough, and fatigue [52], but also cause headache, anosmia, and dysgeusia [8], even disturbance of consciousness and seizures [53], acute myelitis [54]. Sporadic cases had also reported the existence of SARS-CoV-2 in brain tissue [55], which showed its potential involvement in the CNS. Panciani et al. [56] also used the three-phases model to explain CNS invasion by SARS-CoV-2. The model included (i) neuro-invasion via bloodstream or along the olfactory nerve, (ii) decreased viral load via CNS clearance, and (iii) immuno-mediated CNS damage.

The other coronavirus with neuro-invasive potential is SARS-CoV, which caused the SARS pandemic. It has been clear that SARS-CoV is able to infect monocytes-macrophages [31] and dendritic cells [32] and penetrate into the CNS via the hematogenous route. The spread of SARS-CoV to the CNS has been reported both in patients and in animal models. The virus has been proved to exist in the sera and cerebrospinal fluids of two patients [57], and the intranasal infection of transgenic mouse models expressing hACE2 demonstrated the dissemination through the olfactory bulb and the presence of virus in the CNS [58].

Other viruses such as rabies virus and herpes simplex type 1 virus (HSV-1) are both regarded as neurotropic viruses and have a big effect on the CNS. Rabies virus is a highly neurotropic RNA virus, and the glycoprotein of that may have high specific affinity to the nicotinic acetylcholine receptor (nAchR) on neuronal cells [59], the neuronal cell adhesion molecule and the low-affinity nerve growth factor receptor [60]. The existence of these receptors or co-receptors has made the infection of cells complicated. The viral entry into neuronal cells was speculated to happen through various mechanisms, such as retrograde axonal transport [11], nAchR-mediated transcytosis [59], and clathrin- and caveolae-mediated endocytosis [35]. HSV-1 is a neurotropic double-stranded DNA virus. Similar to other neurotropic viruses, it can invade the CNS via two ways (bloodstream and neuronal route) and cause neurodegeneration. In addition to this, MMP in the extracellular matrix would be up-regulated (especially MMP2 and MMP9) [34], and this may lead to the disruption of the BBB and cause edema and hemorrhage [61].

The infection mechanisms of those viruses can be classified as passive diffusion (viruses passively diffuse between endothelial cells); endothelial cell infection (viral tropism is compatible to endothelial cell infection and virus replication in endothelial cells allows for virus release on the basolateral membrane of the endothelium, therefore releasing infectious viral particles toward the adjacent tissue); virus transcytosis (endothelial cells are not infected but still uptake circulating viral particles into non-degradative endosomal vesicles); cell-associated virus transport (viruses infect or are carried by blood circulating cells, which undergo blood-to-tissue transmigration throughout the endothelial cells) [62]. These pathways of entry into the CNS are not mutually exclusive and may vary depending on the immune context or specific virus. The existence of more than one pathway may be used by certain viruses, if possible, in the real model.

## 3. Drug-Loaded Nanocarriers across the BBB

In the past decades, various studies on investigating the ability and efficiency of nanomaterials used as drug carriers to cross the BBB have been reported. Inorganic nanomaterials, such as silica NPs [63], gold NPs [64] and CdSe/ZnS quantum dots [65], have been developed to overcome the BBB. Silica and gold NPs are both regarded as biocompatible material and have shown size-dependent transport efficiency when crossing the BBB, with its efficiency largely decreased as the size increases. Meanwhile, the cytotoxicity of quantum dots should be considered when using them as cargo across the BBB, and the surface modification, such as PEGylation, should be applied to improve their biocompatibility. Synthetic and natural polymeric-based nanomaterials, such as hydroxyl polyamidoamine (PAMAM) [66], poly(D,L-lactide-co-glycolide) (PLGA) [67], and chitosan [68], illustrate their potential as drug carriers because of their high versatility in physical and chemical properties and adjustability in degradation. In addition, lipid-based NPs such as liposomes, with their amphiphilic phospholipid bilayer structure, have shown relatively low toxicity and a high drug-loading capacity [69,70]. Liposome NPs with the surface modified by transferrin, lactoferrin, glucose and glutathione polyethylene (PEG) [70] are proved to be effective strategies to increase the BBB permeability. Therefore, it is very promising to manipulate these materials to enhance the BBB transportation. In this section, the nanomaterials used as drug delivery systems are reviewed and discussed based on different physiological transcytosis mechanisms.

### 3.1. Carrier-Mediated Transcytosis

The blood–brain barrier, formed by brain capillary endothelial cells, is a dynamic interface that takes control of the influx and efflux of numerous molecules between the brain and blood. Not only could this barrier exclude most of the drug molecules but it also possesses several transporter systems, which actively and selectively allows for the passage of desired molecules, including endogenous substances and nutrients, such as peptide, amino acid and glucose [71,72,73,74], which are necessary for brain function and metabolism. These substances can be transported via serval carrier-mediated systems, such as glucose transporter (GLUT), large neutral amino acid transporter (LAT), the monocarboxylic acid transport system (MCT) and glutathione transporter. One of the strategies of crossing the BBB by manipulating carrier-mediated transcytosis is to firstly clearly design and synthesize the structure of new molecules to mimic the nutrient analogues with high affinity to the transporters. Then, these molecules are designed to conjugate on the surface of drug carriers as ligands to overcome the BBB. However, this method is highly dependent on the well-designed structure of the drug, as it is hard to cross the BBB via carrier-mediated transcytosis by simply coupling the drug to another nutrient analogue molecule [5]. Recently, transportation of a drug crossing the BBB via hexose-related transporters has attracted attention. Table 2 lists the designed drug delivery system by using carrier-mediated transcytosis.

**Figure 3:**
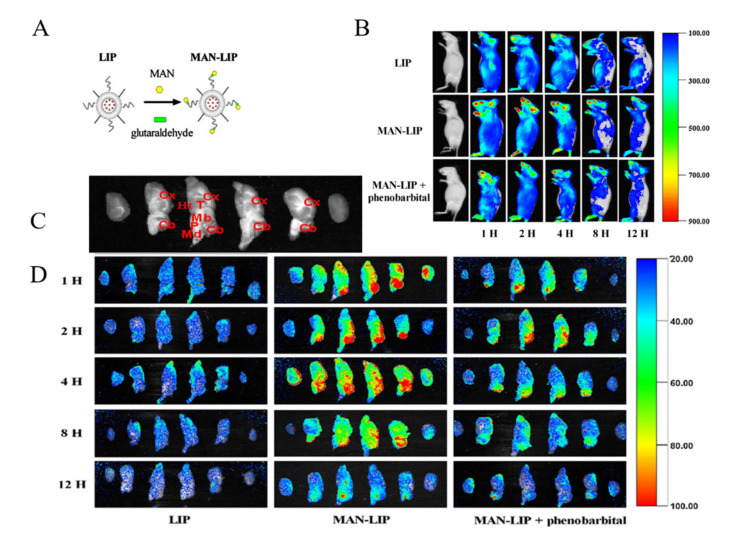
(**A**) Scheme on designing p-aminophenyl-α-D-mannopyranoside (MAN)-conjugated nanoliposome (LIP) drug delivery vehicles (MAN-LIP). (**B**) In vivo time-dependent images of mice after intravenous injection (LIP and MAN-LIP are DiR labeled) of each preparation. MAN-LIP + phenobarbital-represented mice were anaesthetized with phenobarbital (80 mg/kg) following DiR-labeled MAN-LIP. (**C**) The major regions of brain were given as: Cx, cerebral cortex; T, thalamus; Cb, cerebellum; Mb, midbrain; P, pons; Md, medulla; Ht, hypothalamus. (**D**) Ex vivo images of sagittal mice brain sections after intravenous injection of each preparation. (**B**,**D**) The different pseudo colors in the photographs corresponded to the intensity of fluorescence signals. The autofluorescence of the controls was subtracted as the background (reprinted with permission [76]).

**Figure 4:**
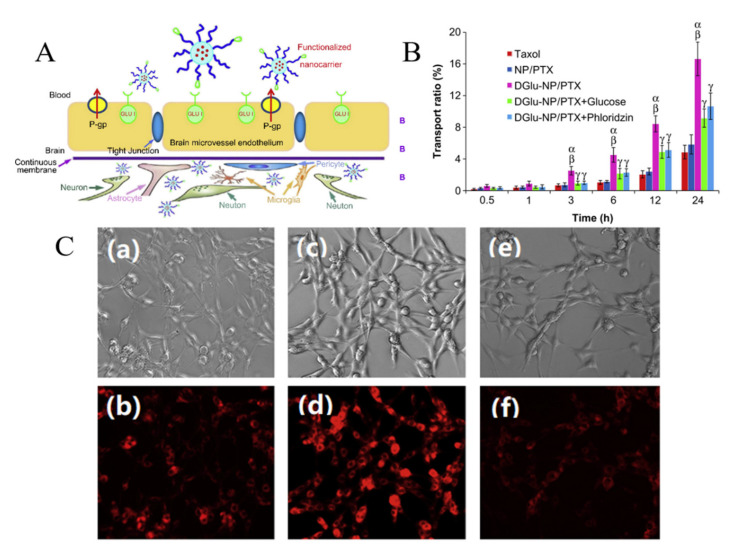
(**A**) Design of 2-deoxy-d-glucose (DGlu) functionalized polymeric (poly (ethylene glycol)-co-poly (trimethylene carbonate)) nanoparticles (DGlu-NP) across the BBB with the assistant of glucose transporter for glioma treatment. (**B**) Transendothelial ability of a drug carrier system with paclitaxel (PTX) loaded on the in vitro BBB model (bEnd.3 monolayer) within 24 h. DGlu-NP/PTX shows the highest transport ratios (%) at each time, and glucose and phloridzin show the competitive effect on this transport pathway. (Data represent mean ± SD (*n* = 3) (α) *p* < 0.01, compared the transportation rate with Taxol; (β) *p* < 0.01, compared with NP/PTX; and (γ) *p* < 0.01, compared with DGlu–NP/PTX) (**C**) Cell uptake (RG-2 cells) by fluorescent microscopy (**a**–**f**) after 60 min of incubation. (**a**,**b**) Rhodamine B isothiocyanate (RBITC)-loaded blank nanoparticles, (**c**,**d**) RBITC-labeled dGlu–NP, and (**e**,**f**) RBITC-labeled dGlu–NP + 20 mm glucose. Concentration of nanoparticles of all samples was adjusted to 300 μg/mL (reprinted with permission [75]).

Glucose is the main energy source for the metabolism of the mammalian brain and this molecule can be transported through the BBB via GLUT, since it cannot be synthesized by brain neurons. GLUT1 is highly expressed as a glycosylated form in the endothelial cells of the BBB [74]. The capacity of the glucose transporter at the BBB is considered to be relatively high since the brain consumes around 30% of total body glucose [88,89]. Recent research proved the feasibility of crossing the BBB via hexose transporters. A simple glucose derivative, p-aminophenyl-α-D-mannopyranoside [76,77], was conjugated on the surface of liposome to study the potential of crossing the BBB. The cell uptake was proved to be enhanced in C6 glioma cells and GLUT1 and GLUT3 overexpressed cells [76] (Figure 3). Although the molecular weight of drugs that can be transported via a carrier-mediated system should not be large, several studies found glucose derivatives with a relatively large molecular weight still capable of crossing the BBB. A series of glycosyl derivatives of cholesterol was synthesized by Wu’s group [80,81,82], with glucose and cholesterol side chains binding to the PEG backbones. These derivatives worked as lipid materials to form a liposome drug delivery system for brain targeting. All of them exhibited the potential of strengthened transendothelial ability, and the derivative former from the PEG with the moderate chain length ($\bar{M_{n}}=1000$) had the strongest brain delivery capacity. In addition, the efficiency of such a transport pathway may be affected when two or more nutrients or its analogues exist because of the competition effect. 2-deoxy-d-glucose conjugated on the poly (ethylene glycol)-co-poly (trimethylene carbonate) nanoparticles (DGlu-NP) showed good BBB penetration and accumulation in glioma cells. However, the transport ratio in vitro model and cell uptake amount of DGlu-NP were obviously lowered with the addition of glucose (Figure 4).

System L, a sodium ion-independent bidirectional transporter, plays a key role in amino acid homeostasis in the brain [90,91]. LAT1 is abundant and selectively expressed on both luminal and abluminal membrane sides of the BBB, and it shows higher substrate affinity than that on peripheral tissues [92,93] and is overexpressed in glioblastoma tumor cells [94,95], which makes the design of the LAT1-mediated drug delivery system possible. Peptide transporters, such as glutathione transporter, are an integral part of the plasma membrane proteins and have been found to be expressed in the brain [96]. In addition, the monocarboxylic acid transport system (MCT), which transports short-chain monocarboxylic acids such as acetic acid, is also essential for brain metabolism [97]. MCT1, a bidirectional transporter for lactic acid and other monocarboxylate compounds, was identified on both the luminal and abluminal membranes of brain capillary endothelial cells [98,99]. Many investigations on manipulating these carriers to design novel drug carriers across the BBB have been conducted. Amino acid and its derivatives, such as phenylalanine and tryptophan derivatives, and glutathione (a tripeptide) are conjugated on the solid lipid nanoparticles (SLNs) [84], Pluronic F127 copolymer NPs [83] and poly-(lactide-co-glycolide) (PLGA) NPs [71,72,85], respectively, to investigate BBB permeability and brain accumulation. β-hydroxybutyric-acid-modified [86] and lactic-acid-modified [87] SLNs are fabricated as well. All of them have been reported to have higher BBB penetration compared to the blank nanocarriers and have higher accumulation in the brain.

### 3.2. Adsorptive-Mediated Transcytosis (AMT)

The initial progress of transcytosis is the uptake of NPs by endocytosis [100]. Endocytosis of cells occurs in two steps: firstly, NPs adhere to the cell membrane, followed by the internalization via energy-dependent pathways [101]. This signifies that the cellular uptake levels are affected by the initial affinity between NPs and the cell membrane. In a physiological environment, the luminal and abluminal surfaces of cerebral endothelial cells are negatively charged [96,102] due to the polarized distribution of carboxyl groups of sialic-acid-containing glycoproteins and sulfate groups of heparan sulfate proteoglycans on the plasma membrane [102]. Some cationic molecules, such as cationized albumin [96,103] and wheat germ agglutinin [104], may have a relatively strong binding affinity for anionic sites on the surface of endothelial cells because of the electrostatic interaction. Based on this, various drug systems have been designed and developed to transport drugs through the BBB by conjugating with cationized molecules, such as chitosan [105,106] and albumin [107,108,109,110], or exerting cationized polymer as a core [35,111,112,113] with various drugs loaded inside and ligands coating the outside. Some cationic molecules, such as cell-penetrating peptide [106] and monoclonal antibody [105], may not only make the conjugation positively charged but also make them applicable for other transport systems across the BBB. However, in this section, we only discuss its potential for AMT.

Poly(propylene imine) (PPI) and poly(ethylenimine) (PEI) are two examples of cationic polymers that are able to condense nucleic acid by ionic interaction and also efficiently deliver genes by escaping from endosomes using proton buffering capacity [35]. The endocytosis of such a drug carrier is completed through AMT, as the capacity of BBB penetration can still be further facilitated by encrusting with oligosaccharide in comparison to the amphiphilic bare one, which can be lowered due to the shielding effect of the anionic PEG layer [35].

In addition to the cationic polymeric core, some polysaccharide can also be used as positively charged materials to enhance the AMT process and more efficiently facilitate the penetrability of the BBB. Maltodextrin NPs have been found to be capable of binding to anionic sites of the cell membrane at an early stage of endocytosis and succeed in penetrating such a barrier via a cholesterol-dependent exocytosis process [112]. Another kind of polysaccharide, chitosan, can also be used to increase electrostatic interaction with cell surface. Chitosan is a promising drug-loading matrix due to its good biocompatibility, degradability, low toxicity, paracellular permeability, strong muco-adhesion and most importantly its polycationic nature [105]. More efficient penetrability and prolonged accumulation of nano-carriers in neuronal cells can be observed after conjugating with chitosan [108]. Similarly, cationized albumin, which is an important nutrient source for cell proliferation, can either be prepared as cationized albumin NPs [113] or applied as shell materials [107,108,109,110] for enhanced cell uptake and transendothelial rate [109,110]. Table 3 provides the major biopolymer-based NPs with a diameter < 150 nm used for the AMT-based drug delivery system.

Furthermore, the zeta potential of the drug delivery system should not be the only factor taken into account when developing a carrier for crossing the BBB; the lipid solubility of the drug carrier is also a vital factor for enhanced AMT. This is because lipid membranes of the endothelium have an innate property to offer an effective diffusive route for lipid-soluble agents [5,111]. Based on this, SLNs, which are biocompatible, biodegradable, non-toxic, and much smaller [109,110], have been used as a drug carrier for crossing the BBB. After the surface of the SLNs was cationized by bovine serum albumin, an enhanced cell uptake and transportation rate similar to that achieved using the drug carriers previously mentioned [109].

However, a positively charged surface is not always good for drug delivery systems designed for crossing the BBB. The cationic surface may represent a higher cytotoxic effect than the neutral counterparts [108,109,110,113,114]. This may be contributed to by the higher amount of cellular uptake and the release of drugs loaded in the carriers, which have relatively high cytotoxicity. In addition to the increased cytotoxicity, such kind of electrostatic interaction by AMT is non-specific, which may result in a random distribution of carriers in cells that can be easily captured by other reticuloendothelial systems, such as the lung and the liver, and the affinity is relatively lower compared to the receptor-mediated drug delivery system.

### 3.3. Receptor-Mediated Transcytosis (RMT)

In contrast to AMT, the binding interaction in RMT has a specific target and has much higher binding affinity between the ligands and the receptors. RMT is initiated by a ligand binding onto its receptor, followed by receptor-mediated endocytosis resulting in the movement of ligand-conjugated drug vehicles across or inside cells. In receptor-mediated drug delivery carriers, the surface of the delivery system is not required to be positively charged but can also be neutral or negatively charged, reducing the potential for increased cytotoxicity. Table 4 summarizes the RMT-based nanomaterial drug delivery system.

**Figure 5:**
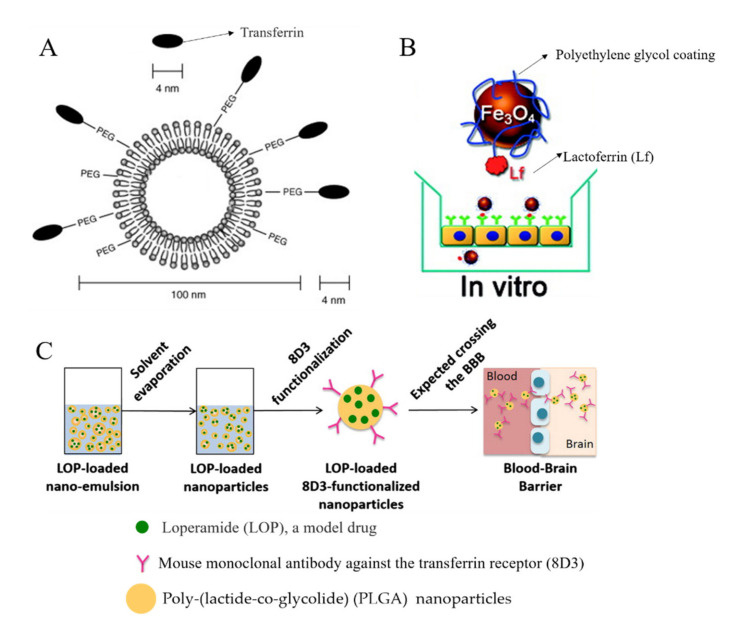
Scheme on the design of ligand-conjugated nanoparticles across the blood–brain barrier (BBB) via transferrin receptor-mediated transcytosis. (**A**). Transferrin (Tf)-tagged pegylated liposome. Tf (black oval) is approximately 4 nm, while a liposome is 100 nm in diameter (reprinted with permission from [116]). (**B**) Polyethylene glycol (PEG)-coated Fe_3_O_4_ with lactoferrin (Lf) conjugated to its surface (reprinted with permission from [119]). (**C**). The process of loperamide (LOP)-loaded nanoparticle preparation from nano-emulsion and its potential use as a nanocarrier system crossing the BBB (reprinted with permission from [118]).

**Figure 6:**
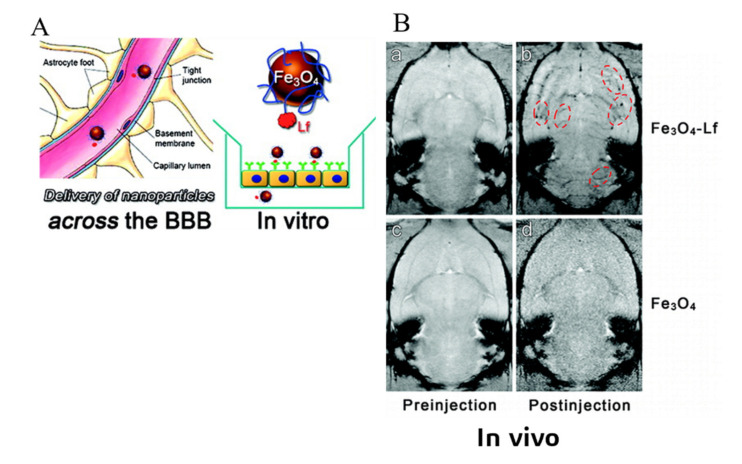
(**A**). The mechanism on lactoferrin (Lf)-tagged magnetic nanoparticles crossing the BBB via injection into the bloodstream. (**B**). Axial T2* images of rat brains captured pre injection and 15 min post injection of Fe_3_O_4_-Lf (**b**,**d**) and Fe_3_O_4_ (**a**,**c**), respectively. The higher contrast of brain blood vessels by the Fe_3_O_4_-Lf probe is highlighted by red dashed-line circles (reprinted with permission from [119]).

Depending on the types of ligands and specific application area, there are various receptors involved in RMT, such as the transferrin (Tf) receptor [105,115,116,117,118,123], lactoferrin (Lf) receptor [119], insulin receptor [121,122], albumin-binding protein [124], low-density lipoprotein receptor [114] and α_v_β_3_ and α_v_β_5_ integrins [120,125]. Transferrin receptor is a transmembrane glycoprotein, responsible for the iron uptake via endo-and exocytosis of Tf [126]. It is expressed widely in the luminal membrane of capillary endothelium [123] and can be found in endothelial cells, epithelial cells, neurons and glial cells in the brain [127]. Based on this, transferrin and transferrin receptor monoclonal antibodies (such as OX26, R17217 and 8D3 [115,118]) are the most commonly used ligands for drug delivery vehicles crossing the BBB. Transferrin-related ligands can be conjugated to various drug-loading matrices, such as human serum albumin NPs [115], pegylated liposome [116], polylactic acid (PLA)-D-PEG [123], and PLGA [117,118] (Figure 5). The penetrability through the BBB of these drug carriers is all enhanced and involves a much faster transportation rate. The transportation process is competitively hindered by free Tf, as it follows the RMT mechanism [123]. Lf, an iron-binding glycoprotein [128], is a member of the transferrin family and can also be applied as a ligand for the transferrin-receptor-meditated transcytosis [119]. The magnetic nanoparticles conjugated with PEG were firstly functionalized by Lf and then injected into the bloodstream of a rat. The magnetic resonance imaging with a higher contrast blood vessel in the brain (Figure 6) presented the ability of Lf-Fe_3_O_4_ nanoparticles across the BBB via the Lf-receptor-meditated pathway. On the other hand, insulin regulates glucose metabolism in the brain and its receptors can be found on the surface of vascular endothelial cells in the brain [127,129]. Pardridge et al. reported that the ligand using the insulin receptor had much higher transport efficacy compared to that using the transferrin receptor [130]; however, Ulbrich et al. found no significant differences in transport efficacy when comparing insulin-receptor-loaded human serum albumin NPs with transferrin-modified NPs or with anti-transferrin-receptor-monoclonal antibodies [122]. Although RMT has the property of specific targeting and higher affinity, a receptor-loaded drug carrier can still have the potential to bind to undesired receptors at different organs. For example, albumin NPs conjugated with low molecular weight protamine is designed to bind to albumin-binding protein (e.g., SPARC and gp60) on glioma and tumor vessel endothelium. However, results from Lin et al. also showed that other reticuloendothelial systems, such as the lungs and the liver, are capable of capturing such albumin-labeled conjugations [124].

**Figure 7:**
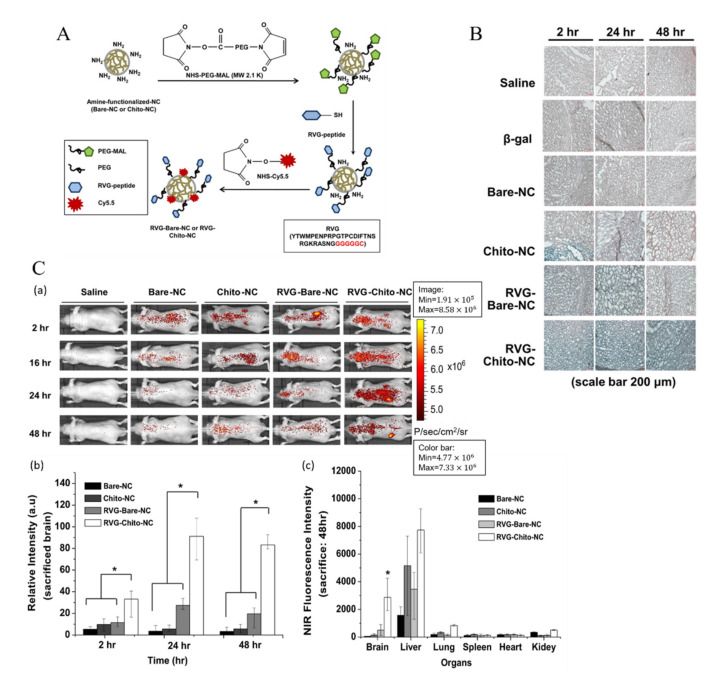
(**A**) Synthesis of rabies virus glycoprotein (RVG29)-cysteamine (Cys) peptide-conjugated Pluronic-based nano-carriers. (**B**) Enlarged images of analysis of β-galactosidase (β-gal) enzyme activity assessed by X-gal staining in the cryosections of the brain. (Bare-NC: blank nanocarriers without chitosan and RVG29 on its surface; Chito-NC: chitosan-modified nanocarriers; RVG-Bare-NC: RVG29 functionalized but without chitosan conjugation; RVG-Chito-NC: nanocarriers with both RVG29 and chitosan modification, obviously showing the strongest β-galactosidase activity.) (**C**) (**a**) In vivo NIR fluorescence images of nude mice after intravenous injection of Pluronic-based nano-carriers (2, 16, 24 and 48 h, respectively). (**b**) Quantification of the different nanocarriers accumulated in the sacrificed brain. (**c**) Quantification of the different nanocarriers distributed in the brain and major organs from the mice at 48 h post injection (*n* = 3, * *p* < 0.01; the intensity of RVG-Chito-NC compared with Bare-NC, Chito-NC, RVG-Bare-NC) (reprinted with permission from [106]).

Furthermore, as mentioned in the AMT section, the cationic surface may favor the adsorption interaction between nanocarriers and cell surface and facilitate the AMT process. However, a positively charged surface is not the only factor that could determine the penetrability of the BBB. There is also a likelihood that drug carriers fail to cross the BBB even if they have relatively strong electrostatic binding to anionic sites [105,120]. For example, unmodified PEG-g-chitosan NPs [105] and bare porous silica NPs [120], both with relatively high positively charged surfaces, still cannot cross the BBB. However, after being conjugated to specific ligands (transferrin receptor and arginine–glycine–aspartate peptide, respectively), receptor-mediated transcytosis plays a dominant role in enhanced BBB penetration. In addition, using AMT and RMT together in a drug carrier system was found to have a synergistic effect on the penetrability of the BBB. A Pluronic-based nano-carrier [106] was designed and studied the synergistic effect of AMT and RMT. The polycation molecule, chitosan, was chosen to facilitate AMT, while the rabies virus glycoprotein, RVG29, which is a cell-penetrating peptide and a ligand for the nicotinic acetylcholine (nACh) receptor, was also functionalized on its surface. Both the X-gal staining images in the cryosections of the brain and in vivo NIR fluorescence images showed that the nanocarriers with RVG29 and chitosan together achieved the highest permeability to the BBB and penetration into the brain (Figure 7).

## 4. Drug Delivery Strategy by the Manipulation Virus

Although many attempts have been made on the synthetic nanomaterial drug delivery systems, those strategies still produce problems, such as particle instability, non-uniform drug release and clearance by phagocytes, and it is still difficult to effectively cross the BBB using the nanomaterial system under the current stage. It is necessary to exploit other mechanisms. Neurotropic viruses could invade the CNS by a cell-type-specific paracellular pathway, by the control of the expression of tight junction proteins (upregulation of MMP2 and MMP9 and/or downregulation of occludin and claudin-5), by a host-response-induced BBB disruption, and/or by a Trojan horse mechanism via monocytes [11,42,62]. Therefore, using those mechanisms may be one of the solutions for targeted delivery to the CNS, and the studies on the combination of the virus-like particle (VLP) and nanomaterials as drug vehicles are attracting more attention (Table 5). VLPs are self-assembled, homogeneous NPs derived from the coat proteins of viral capsids but without their natural genome, which makes them non-infectious.

Based on the neurotropic property of a specific virus and the related mechanisms mentioned above, several non-viral drug carriers and viral vectors have been developed to enhance the penetration of the BBB and transport drug molecules to the CNS. The rabies virus glycoprotein peptide (RVG29) is a peptide with 29 amino acids derived from the rabies virus glycoprotein and has an innate property to specifically bind to nAchR. Because of its high brain penetration capability, several RVG-conjugated drug delivery systems, such as PEI [35], chitosan nanocarrier [106], dendrimers [59] and gold nanorods [131], showed enhanced receptor-mediated transcytosis, higher blood–brain barrier (BBB)-crossing efficiency, and even improved in vivo distribution in the CNS. On the other hand, the permeability and integrity of the BBB can be weakened by decorating related viral protein on the surface of nanocarriers or using a viral vector. The negative factor (Nef) protein of HIV is thought to be essential to HIV-associated immune- and neuroimmune pathogenesis and may target cells of the central nervous system [132]. The delivery of Nef peptides to the BBB in vitro model by magnetic NPs showed reduced transendothelial electrical resistance and disrupted the integrity of the apical blood–brain barrier. Non- or deficient-replication HSV vectors [133] also have a similar effect on impairing the BBB by up-regulating MMP2 and MMP9.

**Table 5:** Strategies on developing nanocarriers by manipulating viruses.

Virus		Design of Drug Carrier	Effects on Crossing the BBB
Rabies Virus	modified rabies virus glycoprotein (RVG)	Poly(mannitol-co-PEI) or chitosan nano carrier as non-viral vector	Enhanced receptor-mediated transcytosis by stimulating the caveolar endocytosis [35,106].
		RVG-conjugated polyamidoamine dendrimers—PEG as gene transporter	A clathrin and caveolae mediated energy-depending endocytosis. Higher blood–brain barrier (BBB)-crossing efficiency [59].
Herpes simplex virus (HSV)	Non/deficient-replication HSV vector	Vector conjugated with Nerve growth factor (injected into cerebrospinal fluid)	BBB score was largely decreased. A gradual limit recovery of motor function [133].
		Vector engineered with vascular endothelial growth factor	Lower infarct volume. Without aggravating cerebral edema. Potent for the therapy of stroke [134].
	HSV type 1 antibody		Possibly plays a protective role in the early stages of AD [135].
HIV	negative factor (Nef) peptide of HIV	Nanomedicine -based delivery	Disrupted the apical blood–brain barrier and reduced transendothelial electrical resistance. Reduced expression of the tight junction protein, ZO-1 [132].
	HIV cell-penetrating peptide Tat	Attached on the exterior of the nanocontainer	More than one uptake mechanism via receptor-mediated endocytic pathways [136].

## 5. Routes of Administration of Nanoformulated Drugs Delivered to the Central Nervous System

Animal studies show that the current forms of the administration of nanoparticle-carried drugs to the central nervous system are: 1. systemic (oral and intravenous) administration, 2. direct brain/intrathecal administration, and 3. transnasal administration. The most convenient route for nano-drug administration is systemic delivery (oral and intravenous). However, the percentage of administered drug reaching the brain via this way is usually below 1–4%, due to the low permeability and the poor BBB selectivity, which means that the remaining 96–99% of the drug is off-target and would be potentially responsible for the systemic side effects, mainly phagocytosed by monocytes and macrophages and accumulated in the liver and spleen [137,138].

The direct delivery of nano-drugs to the CNS, bypassing the BBB and brain–spinal cord barrier (BSCB), is possible by intrathecal injection with direct drug delivery into the cerebrospinal fluid. This route of delivery reveals several advantages over systemic, peripheral administration. It leads to an immediate high concentration of the drug in the cerebrospinal fluid; thus, smaller doses of nano-drugs could be used, thereby minimizing any potential side effects. Importantly, the tightness of the BBB prevents the systemic spread of the nano-drug and significantly limits its penetration from the brain to the general circulation, which effectively reduces its side effects and toxicity. Compared to the freely administered molecules, intrathecal administered nanomedicines are well retained within the central nervous system, and encapsulated payloads experience slower clearance and mixing within the cerebrospinal fluid, which can enhance tissue exposure [139]. The encapsulation of small molecules within colloidal delivery systems offers a number of advantages, such as improvements in drug pharmacokinetics within the central nervous system, reduced toxicity, and enhanced efficacy. Although current clinical work has focused on the development of intrathecally delivered nanomedicine for the treatment of pain, neurodegeneration, and cancer of the central nervous system, it can be expected that this method of drug delivery to the central nervous system will be much more widely used. The clinical experience to date shows that it is a safe and effective method, which justifies a much wider use in the treatment of many central nervous system diseases [139]. The only drawback seems to be the invasiveness of this method. For diseases requiring chronic treatment, the need for multiple lumbar punctures is burdensome for the patient and may limit the frequency of using this method in the treatment of central nervous system diseases.

The intranasal route of drug delivery offers a unique opportunity for the delivery of pharmaceutically active ingredients (APIs) to the central nervous system. It is the less invasive route of drug delivery compared to the intrathecal administration method, and it already has been used successfully in clinical trials, showing improved cognition after intranasal insulin application in Alzheimer’s disease patients [140,141,142]. Intranasal drug delivery enables both small and large molecules to bypass the BBB via the nerves of the nasal cavity: the olfactory and trigeminal nerves towards the posterior region of the brain [141]. Importantly, the olfactory neuroepithelium is the only region of the central nervous system that is not protected by the BBB; thus, it is in indirect contact with the external environment. Consequently, it becomes a unique access port to the brain [139]. The olfactory and trigeminal nerve pathways provide brain delivery via either a slow intracellular axonal transport (hours or even days) or a fast perineural paracellular transport (minutes) from the sub-mucosal space to the cerebrospinal fluid compartment. A small portion of the drug administered into the nasal cavity also enters the general circulation, and then it can reach the brain after crossing the BBB [140,141]. Since only a small amount of the drug can be absorbed from the olfactory mucosa into the blood after standard nasal administration, it is generally accepted that systemic toxicity and systemic pharmacokinetic issues can be omitted in this route of administration, importantly, as the drug does not reach the liver or undergo biotransformation in the liver, and therefore it does not show a first-pass effect.

Notwithstanding the significant advantages of transnasal drug delivery routes, the proper formulation of drugs prepared for administration remains an important challenge, especially for drugs with adverse physicochemical and biopharmaceutical properties, such as rapid chemical or enzymatic degradation, poor moisture solubility, and low permeability. It requires a formulation capable of increasing drug transport to the brain, without interfering with the structure and physiology of the nasal epithelium. Pharmaceutical nanotechnologies are of strategic importance for developing the formulation of these substances for transnasal drug delivery to the brain. Nanomedicines could further contribute to making nose-to-brain delivery a reality.

The potential limitations of the method are the small volume of the nasal cavity, which limits the amount of formulation that can be administered this way, poor access to the olfactory region using conventional nasal application devices, the short residence time of the drug in the nasal cavity before it is removed outside, low hydrophilic bioavailability, and possible mucosal irritations [141]. In the near future, both the improvement of devices for applying larger volumes of drug nano-formulation to the upper nasal cavity and modification of the nanomedicine surface, e.g., with mucus-penetrating particles, penetration-enhancing agents, lectin-modified nanocarriers and cell-penetrating peptides (the last two promote translocation of the carrier into the central nervous system), seem to be key strategies for optimizing drug delivery from the nasal cavity to the brain [141]. Therefore, almost every nanocarrier of the drug has been tested for nasal delivery to the brain, as listed in available review articles [141].

Comparison of the brain targeting of olanzapine loaded PLGA NPs with a free drug in a solution, administered both intravenously and intranasally to sheep, showed that the uptake of NPs into the brain was 6.35 and 10.86 times higher, respectively. Noteworthy is the significant advantage of the nasal route of nanomedicine administration in increasing olanzapine transport to brain tissue [143]. Nimodypine-loaded microemulsions administered intranasally to rats were rapidly absorbed into both the general circulation (tmax = 1 h) and the brain, with the concentration of the drug in the olfactory bulb being three times higher after intranasal administration than after intravenous nano emulsion of this drug [144]. NPs play a special role for targeting drugs to the brain due to their great potential for the transport of many drugs to the brain that are normally unable to cross the BBB. The current results show that polysorbate 80-coated PLGA NPs significantly transported the drug donepezil in comparison with the free drug solution to the brain. The high concentrations of donepezil achieved in the brain may be a significant improvement for treating AD.

Quantitative data analysis in 73 publications from the last 30 years on the transnasal route of drug administration revealed such large differences in work results that it was impossible to establish a correlation between the physicochemical properties of drugs and their formulas and the effectiveness of targeting in the brain. The only regularity that can be directly compared was the effect of the drug form on its transport from the nose to the brain. The percentage of drugs reaching the brain from various formulations shows that drugs encapsulated in particles reach the brain in a greater amount (60%) than the free drug administered intranasally in solution (36.6%), and it should be noted that 24 of the 32 compared particulate drug formulations had a size of 50–200 nm [145].

Undoubtedly, the transnasal route of administering the drug’s nano-formulation directly to the brain tissue still requires a lot of research, especially the standardization of nanomedicine preparation methods, the functionalization of NPs, arming them with directional particles that improve transport to the brain, etc. This is a prerequisite for obtaining more reproducible, more comparable results of experiments in vitro and in vivo on laboratory animals [145]. On the other hand, this route of drug delivery to the central nervous system has unique advantages, such as ease of administration, non-invasiveness, rapid onset of the drugs’ action, a relatively permeable absorption surface, reduced enzymatic activity and the avoidance of hepatic first-pass metabolism [141]. Undoubtedly, this method promises significant progress in the pharmacological treatment of many neurological diseases, and it seems that the best is yet to come [141,142].

Regardless of the path that nanomedicines have used to reach the brain, it is necessary to better understand the effects of the nano-formulation building elements themselves on neurons as well as on glia cells. Specifically, chronic toxicity to the central nervous system, as well as the immunogenicity of nanocarrier components, must be evaluated in detail, especially for medications anticipated for long-lasting treatment [137,138,146]. In fact, the main part of the NP drug contains a polymer, and the drug load is usually about 10% by weight of the NPs; the remaining 90% consists of a polymer and another functional component of the NP molecule. When NPs are repeatedly administered in a long-term treatment mode, cells and tissues are constantly exposed to the chemical component of the NPs. In addition, the pharmacokinetics and tissue clearance of API and NP carrier can differ significantly. For example, in rat studies, it was shown that the drug loperamide remained in the brain for several hours, while the PLGA NP carrying it disappeared from the brain only after about 24 h [146]. Therefore, their effects on targeted and non-targeted neighbor cells should be checked to exclude possible toxic and immunogenic effects [146]. It can be expected that the improvement in the efficiency of the NP structure—greater loading API in proportion to nanocarriers—should result in a better drug effect, meaning less long-term therapy and therefore lower exposition of brain cells on polymers and other components of nano-formulation.

## 6. Conclusions

The complex structure of the BBB allows for the extensive filtration of materials for the protection of the brain and CNS. Although such filtration is necessary, it leads to difficulties in delivering drugs to treat various CNS diseases. The investigation of the physiological transport and viral-induced BBB breakdown mechanisms has provided innovative strategies for overcoming the BBB. The nanomaterial-based drug delivery systems functionalized with endogenous substances and essential nutrients for the brain, as well as their derivatives, are able to penetrate the BBB and deliver drugs to the brain via CMT. However, the molecules conjugated on their surface, which can be transported by specific carriers, must be well designed. Free endogenous substances and nutrients may have a competitive effect with such a drug carrier system and lower the drug transport efficiency. In addition, drug nano-carriers with cationic molecules and ligand modified on their surface both illustrate higher BBB permeation and brain accumulation via AMT and RMT, respectively. The binding affinity between the ligand and receptor pair for RMT is stronger than it is for AMT, and the binding sites are specific, while AMT drug delivery carriers have a higher binding capacity and the inhibition by saturation seldom happens. Furthermore, drug transport system coupling dual functional molecules (manipulating AMT and RMT together) were found to have a synergistic effect on crossing the BBB. In addition to the drug carriers based on the physiological BBB transporting mechanism, the studies on exploiting viruses with nanomaterials as drug carriers are under investigation and still at an early stage. However, drug carriers combining VLP and the functional proteins on the viruses are proved to be effective for the BBB transportation.

To conclude, nanomaterials have proven their ability to cross the BBB and their potential in drug delivery systems involving the BBB. Further studies investigating the synergistic effects of CMT, AMT and RMT in BBB drug delivery and the identification of compatible nanomaterial–drug pairs would allow for a wider range of applications in the diagnostic imaging of CNS diseases and effective treatment through drug delivery systems. The further rapid development of pharmaceutical nanotechnology and the improvement of direct drug delivery to the brain that bypasses brain barriers promise a breakthrough in the near future in the treatment of many nervous system diseases, including inflammatory, autoimmune and mental diseases, as well as neurodegeneration and brain cancer.

## Figures and Tables

**Table 1:** Viral disruption of the BBB and its effects on BBB transportation.

	Virus	Effects on CNS
Flaviviridae	Hepatitis C virus (HCV)	Human brain endothelial cells express functional receptors that support HCV entry and replication; HCV infection promotes endothelial permeability and cellular apoptosis [19].
	West Nile virus (WNV)	Increase activity and mRNA expression of matrix metalloproteinases (MMP) 9 in mouse brains; a Trojan horse mechanism [20].
	Japanese encephalitis virus (JEV)	Increase MMP9 expression in a reactive oxygen species (ROS)-dependent manner [21].
	Dengu Virus	Mediated via the release of histamine by a virus-induced cytokine.
	Zika Virus	Downregulation of occludin and claudin-5 levels [22]. A cell-type-specific paracellular pathway to cross the placenta monolayer [23].
coronavirus	SARS-CoV-2	Spike protein S1 binding to ACE2 [24,25,26]. Much higher affinity [27].
	HCoV-OC43	Neuronal retrograded (olfactory bulb) [28] and hematogenous pathway [29]; May have neuronal degeneration [28].
	HCoV-229E	Invasion via the circulation of bloodstream [28]. Neuro-invasive under immune-suppressed environment [30].
	SARS-CoV	ACE2 receptor. Both hematogenous route [31] and olfactory bulb [32];
Other viruses	HSV	Bloodstream and neuronal route [33]; Up-regulate MMP2 and MMP9 and disrupt BBB [34];
	Rabies virus	Rabies virus glycoprotein as brain-targeted ligand andthe nicotinic acetylcholine on neuronal cells as receptor [35];
	MAV-1	Stimulate an innate host response to induce BBB disruption [36]; Possible invasion by a Trojan horse mechanism via monocytes [37,38];
	Theiler’s murine encephalomyelitis virus	Induce acute encephalitis with alterations in tight junction protein expression [39].

**Table 2:** Drug delivery systems by carrier-mediated transcytosis (hexose, amino acid, peptide and monocarboxylic acid transporters) and their effects on crossing the blood–brain-barrier (BBB) and brain.

The Compounds on the Carriers’ Surface		Drug Carrier	Transport Pathway	Effects on BBB and Brain
Hexose derivatives	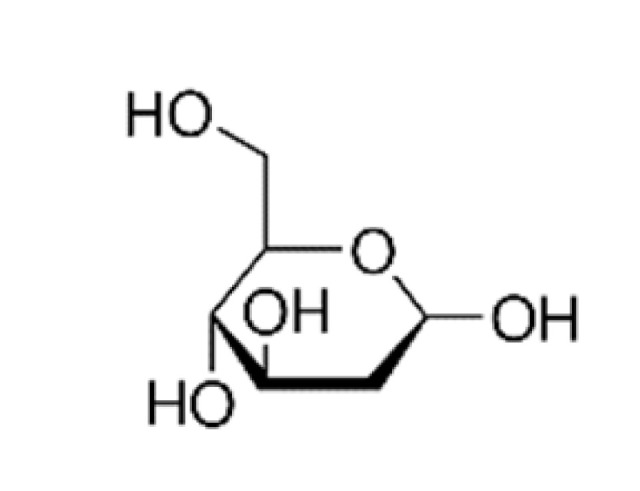	Poly(ethylene glycol)-co-poly(trimethylene carbonate) nanoparticles	GLUT1 [75]	Higher internalization amount by glioma cells. Successful penetration of the BBB. Showing specific and efficient accumulation in intracranial tumor.
	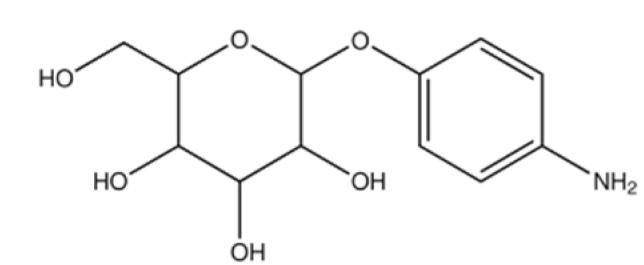	Liposomes	GLUT1 and GLUT3 [76,77,78]	Enhanced cellular uptake and accumulation in the brain. Stronger transendothelial ability.
	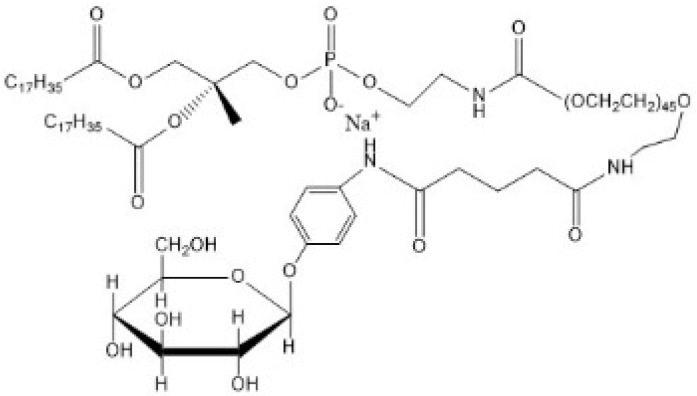		GLUT1 [79]	Long circulation in blood. Less leakage in the blood component-containing system. Efficacies in killing glioblastoma cells.
	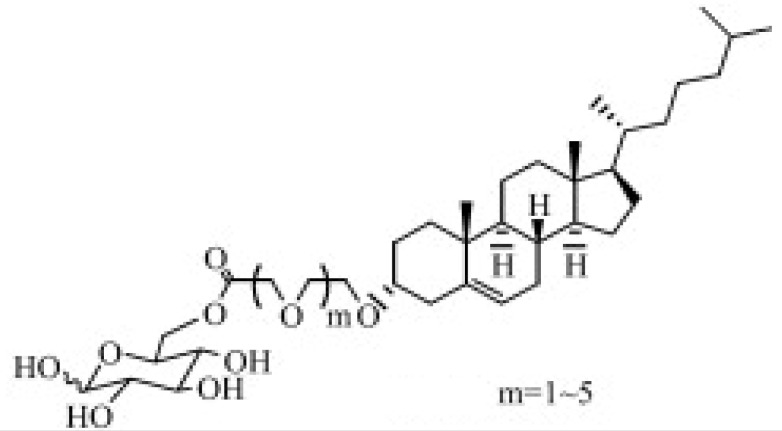		GLUT [80,81,82]	The potential of brain targeting. Molecules with moderate chain length exhibiting the strongest brain delivery capacity.
Amino acid derivatives	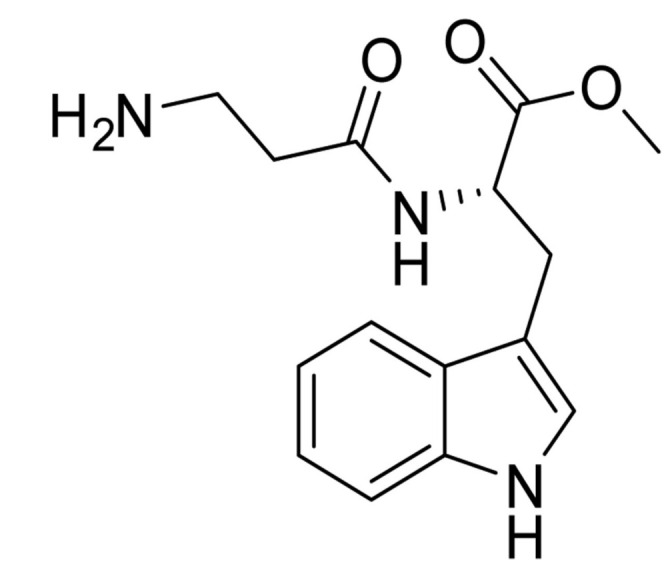	Pluronic F127 copolymer nanoparticles	LAT1 [83]	Successful drug delivery to the hippocampus in the brain. Increased tryptophan uptake at epileptogenic focus.
	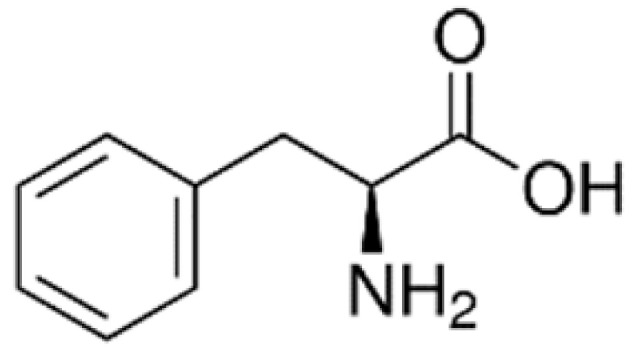	Solid lipid nanoparticles (SLNs)	LAT1 [84]	Higher accumulation in the brain.
Peptide	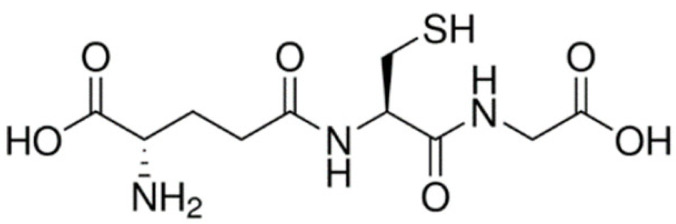	Poly-(lactide-co-glycolide) (PLGA) nanoparticles	Glutathione transporter [71,72]	Higher BBB permeation and brain uptake. Not substrates of P-glycoprotein (P-gp) and not being effluxed by P-gp.
		Poly(ethylene glycol)ylated PLGA	[85]	
Monocarboxylic acid	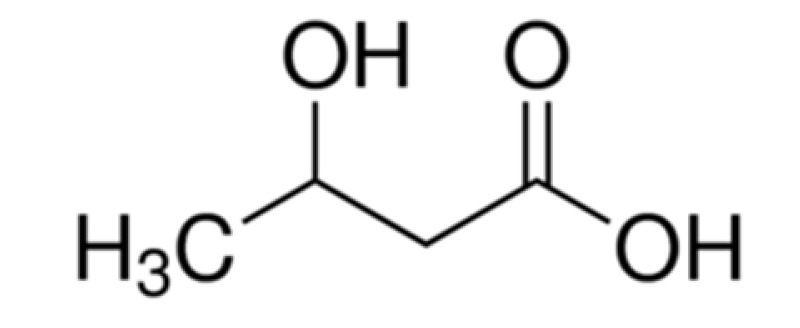	SLNs	Monocarboxylic acid transport system (MCT) 1 [86]	Improved brain uptake.
	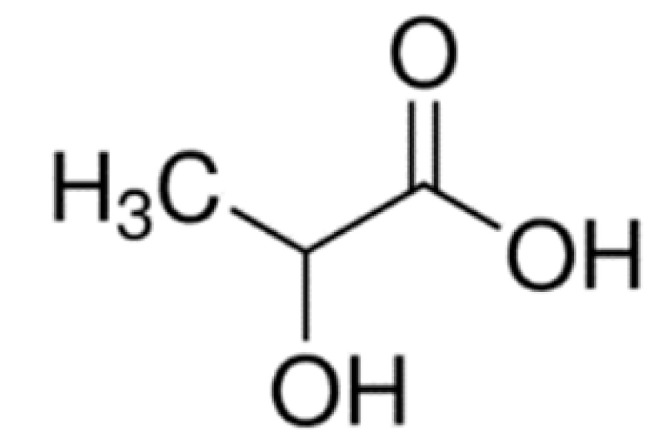		MCT [87]	Selective brain uptake.

**Table 3:** AMT-based nanomaterial drug delivery system.

Drug Carrier	Modification Methods	Zeta Potential
poly(propylene imine) (PPI) [111]	Oligosaccharide-modified	Positively charged
Poly(ethylenimine) (PEI) [35]	Conjugating with rabies virus glycoprotein and PEG	Relatively neutral
Maltodextrin nanoparticles [112]	\	25 ± 1.5
PEG-g-chitosan [105]	Transferrin receptor monoclonal antibodies (OX26)	23.0 ± 0.4
Solid lipid nanoparticles (SLNs) [109,110]	Conjugating bovine serum albumin	10.3 ± 0.6
Pluronic-based nano-carrier [106]	Chitosan and rabies virus glycoprotein-conjugated	12.1 ± 0.8

**Table 4:** RMT-based nanomaterial drug delivery system.

Drug Carrier	Ligand	Receptor
Human serum albumin nanoparticles [115]	Transferrin (Tf)/ transferrin receptor monoclonal antibodies (OX26 or R17217)	Transferrin receptor
Pegylated liposome [116]	Transferrin (Tf)	
Poly-(lactide-co-glycolide) (PLGA) [117]		
PLGA [118]	Mouse monoclonal antibody against the transferrin receptor (8D3)	
Polyethylene glycol (PEG)-coated Fe_3_O_4_ [119]	Lactoferrin (Lf)	Lf receptor
Nanoliposomes [114]	Apolipoprotein E (ApoE)-derived peptides	Low-density lipoprotein receptor
Pluronic-based nano-carrier [106]	Rabies virus glycoprotein	g-aminobutyric acid (GABA) /nicotinic acetylcholine (nACh) receptor
Poly(ethylenimine) [35]		
Porous silica nanoparticle [120]	RGD (arginine–glycine–aspartate) peptide	α_v_β_3_ and α_v_β_5_ integrins
Glial-derived neurotrophic factor [121]	Chimeric monoclonal antibody	Insulin receptor
Human serum albumin (HSA) nanoparticles [122]	Anti-insulin receptor monoclonal antibody (29B4)	
